# Evaluating the reproductive ability of breeding rams in North-Eastern Spain using clinical examination of the body and external genitalia

**DOI:** 10.1186/s12917-015-0600-9

**Published:** 2015-11-26

**Authors:** René Mozo, Ana Isabel Galeote, José Luis Alabart, Enrique Fantova, José Folch

**Affiliations:** Unidad de Producción y Sanidad Animal, Centro de Investigación y Tecnología Agroalimentaria (CITA), de Aragón, Av. Montañana 930, 50059 Zaragoza, Spain; Oviaragón-Grupo Pastores, S.C.L. Mercazaragoza, Camino Cogullada s/n, 50014 Zaragoza, Spain; Instituto Agroalimentario de Aragón – IA2 - (CITA-Universidad de Zaragoza), Zaragoza, Spain

**Keywords:** Breeding soundness evaluation, Sheep reproduction, Ram clinical examination, Sheep breeding, Rasa Aragonesa, Risk of failure

## Abstract

**Background:**

Predicting the ability of rams to detect, mate and fertilise ewes in oestrus accurately is certainly difficult; however, tests based on clinical examinations have been performed to assess the overall potential capacity of rams to serve and impregnate ewes. Clinical examinations for breeding soundness evaluation were carried out in 897 Rasa Aragonesa (RA) rams from 35 flocks in North-Eastern (NE) Spain. Clinical examinations of head, trunk, limbs and genitals were performed in each ram. Blood samples were collected for a serological study of *Brucella ovis*. The sheep owners were surveyed regarding the characteristics of the flock, rams’ health history and the management of rams. The clinical alterations found were classified according to severity (mild or severe). Rams were classified as suitable (without lesions or with only mild lesions) or unsuitable (with severe lesions) for breeding depending on the results of the clinical examinations.

**Results:**

The results showed that 60.6 % of rams presented some type of alteration (mild: 43.3 %; severe: 17.3 %) in various body parts (genitalia: 31.6 %; head and trunk: 37.2 %; limbs: 15.5 %), and that 16.7 % of rams were classified as unsuitable breeders. The most common genital alterations were ulcerative posthitis (18.7 %) followed by testicular lesions (5.3 %). The highest prevalence of unsuitable breeders was found in the category of adult and aged rams (13.8 % and 37.4 %, respectively) and in the category of emaciated rams (33.3 %). All rams examined were seronegative to *Brucella ovis*. The mean percentage of rams in flocks was 2.8 % (min: 1.6 %; max: 4.6 %); nevertheless, this percentage dropped to 2.5 % (min: 1.4 %; max: 3.7 %) and 2.1 % (min: 0.3 %; max: 3.5 %) when only suitable or effective (suitable mature) rams were considered.

**Conclusion:**

Thus, it is concluded that there are fewer effective rams in farms than farmers realise. Frequent clinical examination of males is recommended in order to identify potentially infertile rams.

## Background

Reproductive performance is the most important parameter affecting flock profitability [1], in which the reproductive capacity of the rams plays a key role. In fact, fifty percent of the reproductive potential of a flock is provided by the ram [2]. It has been proposed that one of the most useful services a veterinary practitioner can offer to sheep owners is a regular fertility evaluation of rams [3, 4]. Unfortunately, there is no test available that makes it possible to accurately predict a ram’s ability to detect, mate and fertilize oestrous ewes [5]. Tests have been performed to assess the overall potential capacity of rams to serve and impregnate ewes, also known as breeding soundness evaluations (BSE). A BSE may include anatomical and structural examinations, and assessment for health status, body condition score (BCS), testicular measurements, semen quality, libido and specifically *Brucella ovis* analysis [6]*.* There are technical difficulties in performing systematic and generalised, time-consuming analyses for sperm quality [6] and libido assessment using serving capacity tests (SCT) in commercial flocks [7]. Otherwise, most of the problems adversely affecting the reproductive ability of rams can be easily detected through a careful physical examination [3]. High prevalence of physical alterations in rams is related to decreased flock production. Clinical examinations have been proposed to detect potentially infertile or sub-fertile rams according to physical characteristics related to their breeding capacity [2, 5, 8]. These tests, based on physical evaluations, are easy to perform and do not require technical material or specialised support. In addition, it has been reported that it is possible to progressively reduce the rejection rates of males adopting BSE as a routine [9]. Various programmes including this kind of test have been performed in several countries, including Greece, the United Kingdom, New Zealand and Australia [5].

In addition to BSE physical examinations, blood collection in order to test rams for infectious agents could be easily performed, and does not take an excessive amount of time, although laboratory support is needed. Some infectious agents that can affect ram fertility are *Brucella ovis, Histophilus ovis*, *Actinobacillus seminis*, and *Corynebacterium pseudotuberculosis* [6, 10, 11]. *Brucella ovis* is the main agent causing ram epididymitis [11]. The identification of positive rams would prevent pathogen transmission.

Breeding soundness evaluations should be performed at least two months before breeding [5] to allow sheep owners to recover rams from pathologies or poor physical conditions. Such a period of time is required for sperm production and maturation. It is essential to record conditions that should be corrected in order to enable the ram to perform to its maximum reproductive ability, as well as to detect infections that could be transmitted to ewes in the breeding season [3]. Appropriate feeding during the pre-breeding period is essential owing to its close relationship to sperm production and the sexual activity of rams [12, 13], while taking into account the mantra *“fit but not fat”* [12].

Due to the importance of rams’ fertility for the productivity of the flocks, in Spain, the cooperative society of ovine producers, Oviaragón–Grupo Pastores, in collaboration with the Centro de Investigación y Tecnología Agroalimentaria de Aragón (CITA) have developed a programme to assess the breeding soundness of rams serving in commercial flocks as part of the health management of the associated sheep farms. In the present study, we describe the results of this programme, which is based on the physical examination of Rasa Aragonesa rams managed in semi-extensive natural breeding systems. We will describe the prevalence of clinical alterations in the rams examined. Next, we will present the results of surveys of sheep owners about the general management of rams, the characteristics of flocks, and the selection criteria for ram lambs as future breeders, and the main reasons for culling rams. In the future, our objective will be to associate the prevalence of alterations and the management of rams with the fertility records of commercial farms.

## Methods

### Animal ethics

Authors declare that the present study was performed in compliance with the Directive 2010/63/EU of the European parliament and of the council of 22 September 2010 on the protection of animals used for scientific purposes. All the animals included were submitted to non-experimental, clinical veterinary practices or practices not likely causing pain, suffering, distress or lasting harm equivalent to, or higher than, that caused by the introduction of a needle in accordance with good veterinary practices. All these procedures were aproved by the *Comité Ético de Experimentación Animal del Centro de Investigación y Tecnología Agroalimentaria de Aragón.*

### Animals

The study was carried out in 35 commercial flocks managed in semi-extensive conditions, mainly located in Aragón and associated with the *Oviaragón–Grupo Pastores* cooperative society. There were evaluated 897 Rasa Aragonesa (RA) rams managed as natural breeders which were evaluated. The prevalence of alterations was more deeply studied in rams older than 12 months (*n* = 774). Rams were individually evaluated for physical soundness only once.

Rams’ owners were properly informed about the examination methodology. Examination of rams was only performed after the owners’ permission was obtained.

### Clinical examination and standardisation of the evaluation methodology

Physical examinations were performed visually and by palpation. The data were recorded from March to December 2012. The alterations were classified as severe or mild, taking into account the potential detrimental effect on the wellbeing or the reproductive activity of rams. In order to perform a homogeneous and unbiased evaluation, a “clinical protocol” was developed, in which evaluation methodology, criteria for classification of alterations as severe or mild in each body area, as well as treatments were clearly specified. The clinical examination methodology, the classification of alterations and recommended treatments according to severity were based on studies in the literature [3, 8, 14] and were agreed with the 25 veterinarians comprising the technical team of the cooperative.

#### General clinical examination

Firstly, physical soundness was assessed in the standing position, paying attention to general health (digestive disorders such as diarrhoea and meteorism, breathing distress, etc.), the hip joint, the gait and the ram’s conformation. Blood samples were collected for the serological study explained thereafter. The lumbar region of the lambs was considered, to assess the BCS [15] adapted for RA animals [16]. The BCS was assessed at 0.25 intervals and was agreed between two veterinary practitioners.

The rams were restrained by their rump and the rest of the body was carefully examined. Firstly, the rams were evaluated for head alterations (i.e., nasal and ocular discharge, corneal opacity and missing teeth). Secondly, the neck and trunk, mainly the breastbone and the costal area, were checked for sores, wounds, dermatitis, nodules and the presence of umbilical hernia. Finally, the limbs were examined for evidence of inter-digital dermatitis, arthritis or poor hoof integrity. If necessary (excessive development of hooves), hooves were trimmed after examination of the limbs.

#### Clinical examination of the genitalia

Testicular evaluation was performed in the standing position. Firstly, the scrotum was examined for pathological findings such as dermatitis, oedema, nodules and wounds, then the scrotal bag was palpated from the inguinal area to the *cauda epididymis*. The spermatic cord and the *pampiniform plexus* were palpated to assess the presence of scrotal hernia, inflammation of the inguinal lymph nodes, nodules and lesions similar to varicocele. The testes were palpated for consistency, presence of nodules or abscesses, symmetry, free movement inside the scrotum and size (i.e., orchitis, atrophy or testicular hypoplasia). When small testicular size was found, no difference was determined between hypoplasia and atrophy. Finally, the whole length of the epididymis was examined (i.e., epididymitis, atrophy, nodules, etc.).

The prepuce examination was carried out when restraining the ram at its rump. The internal preputial mucosa and the external preputial skin were evaluated, looking for wounds, abscesses, dermatitis, sores (ulcerative posthitis) or abnormal discharge. Special attention was paid to the preputial orifice for ulcerative posthitis or previous preputial blowfly myiasis. The glans and the urethral process were assessed for alterations (wounds, ulceration, inflammation or necrosis). When the glans was extruded, particular attention was paid to difficulty passing through the preputial orifice. The technique to extrude the glans consisted of grasping the sigmoid flexure firmly between the index finger and the thumb, pushing upwards towards the prepuce orifice and, at the same time, pushing the prepuce downwards [8]. In order to simplify the data recording, lesions found in the prepuce were referred to as posthitis while lesions found in the glans were termed balanitis.

#### Serological study

A serological study for *Brucella ovis* (Contagious epididymitis) was performed in all rams examined. Blood was collected in non-heparinized vacuum tubes (Vacuette®; Greiner bio-one, Inc., Frickenhausen, Germany). The samples were sent at room temperature to the laboratory within 48 h of blood collection. The serological analysis was conducted at the Laboratorio de Sanidad Animal del Servicio de Seguridad Agroalimentaria del Gobierno de Aragón (Zaragoza, Spain) using the immunogel diffusion technique, according to WOAH [11, 17].

### Classification of rams

#### Ram classification groups for data analysis

The rams were grouped in two independent ways to study the effect of age and BCS on the clinical examination results: 1) the rams were categorised into four lots according to their age, similar to that of Van Metre et al. [18]: <12: ram-lambs; 12 – 24: yearlings; 24 – 60: adult rams; >72 months: aged rams; 2) the rams were categorised into four groups according to their BCS (<2.5: emaciated; 2.5 – 2.75: low BCS; 3 – 3.5: medium BCS; >3.5: high BCS). Only the rams aged ≥12 months were considered for BCS classification.

#### Ram classification for breeding ability

After each clinical examination and the serological results for *B. ovis*, rams were classified as suitable or unsuitable breeders, according to the severity of the alterations recorded. Rams without lesions or exhibiting only mild lesions were classified as suitable breeders, and rams showing severe alterations were classified as unsuitable breeders.

A lack of 2 to 4 teeth was not considered a severe alteration when the ram had similar BCS to the rest of the rams. In these cases, regular monitoring of the BCS of these rams was recommended in order to remove them when the BCS or testicular size was affected.

Within the group of unsuitable breeders, rams with severe alterations permanently affecting their potential fertility, reproductive activity, or genetic aetiology (i.e., potentially hereditary alterations such as entropion, prognathism and umbilical or scrotal hernia) were classified as “recommended for culling”; otherwise, they were classified as “rams not recommended for breeding in the near future” (who may potentially recover in the future). In such cases, additional evaluations were recommended to assess their recovery. Rams with mild or severe alterations that were not recommended for culling were treated until recovery according to the “clinical protocol”.

### Ram rates in flocks

After clinical examination, the percentages of total, suitable and effective rams in flocks were calculated. The proportion of effective rams for each flock was calculated as follows:

$$ \mathrm{Effective}\kern0.5em \mathrm{rams}=\frac{\mathrm{Number}\kern0.5em \mathrm{of}\kern0.5em \mathrm{rams}\kern0.5em \left(\ge 12\mathrm{months}\right)\kern0.5em -\kern0.5em \mathrm{Unsuitable}\kern0.5em \mathrm{rams}\kern0.5em \left(\ge 12\mathrm{months}\right)}{\mathrm{Total}\kern0.5em \mathrm{number}\kern0.5em \mathrm{of}\kern0.5em \mathrm{ewes}\kern0.5em  and\kern0.5em  rams}\times 100 $$

The risk of failure (probability to find unsuitable rams aged ≥12 months in the flock) was calculated as the number of unsuitable rams divided by the number of the total rams evaluated.

### Sheep owner survey and flock characteristics

Sheep owners were surveyed about the management of the rams, total number of ewes, mean age of rams at first breeding, ram and ewe turnover, and the pathological history of rams. Farmers were also asked to score the main criteria used for replacement of rams (consanguinity, genealogy, genital characteristics, growth rate until weaning, libido, morphology and scrapie resistance genotype). The final section asked for the main reasons for rams culling (age, aggressiveness, BCS, genital lesions, limb lesions, illness and loss of libido). Each criterion was scored from 1 (strongly disagree) to 5 (strongly agree), similarly to that described by Uthlaut et al. [19].

In this survey, the following flock characteristics were added: the mean age and BCS of rams, ram:ewe ratio, percentages of total, suitable and effective rams in the flock, and the risk of failure.

### Statistical analysis

Statistical analyses were performed using SPSS 15.0 for Windows (SYSTAT Software Inc., California, USA). Comparisons of the percentages of rams with alterations (none, mild or severe) between categories in both grouping methods (age or BCS) were performed via a Z-test, using the Bonferroni correction for multiple comparisons. Values were significantly different when *p <* 0.05.

A descriptive analysis was performed for variables evaluated in flocks, such as: the total number of animals, the ram:ewe ratio, the percentage of total, unsuitable and effective rams, the mean age of rams, the mean age at first breeding, the mean BCS of rams, the ram and ewe turnover, the prevalence of rams with alterations and the risk of failure.

## Results

The proportion of rams according to age was as follows: ram lambs 13.7 % (123), yearling rams 17.1 % (153), adult rams 54.6 % (490) and aged rams 14.6 % (131).

**Fig. 1:**
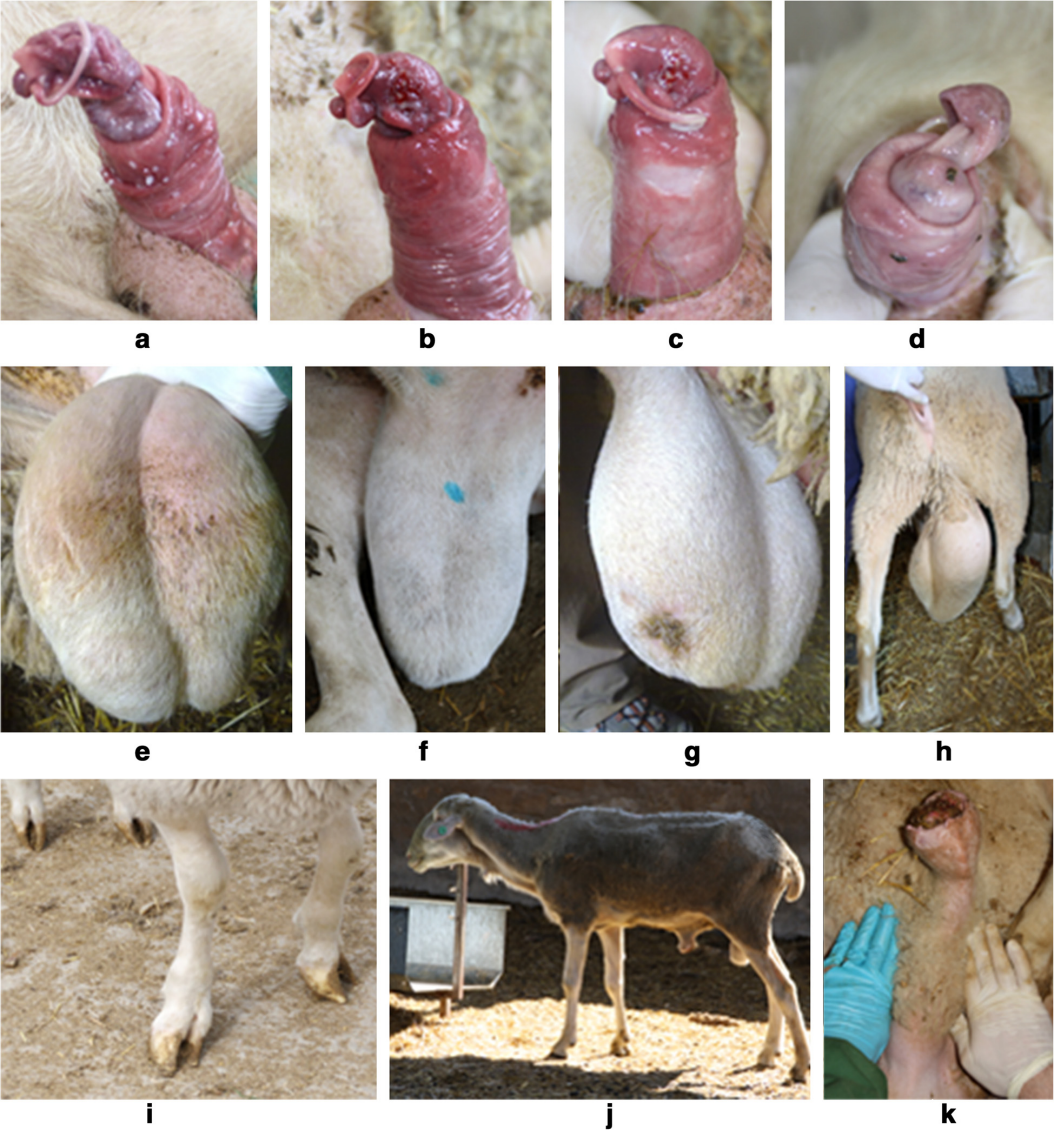
Genital alterations found during clinical examinations: Glans inflammation and ulcerative balanitis (**a**-**b**); Ulcerative balanitis and necrosis in the vermiform appendix (**c**); Anatomical alteration of glans, the head of the glans is partially separated from the body of the glans (**d**); Epididymitis (**e**); Varicocele (**f**); Orchitis and scrotal wound with purulent exudate (**g**); Scrotal hernia (**h**); Alterations of limbs conformation (**i**); Emaciated ram (**j**); Ulcerative posthitis and urolithiasis (**k**)

Three hundred and five of the 774 rams older than 12 months (39.4 %) presented no alterations after the clinical examination, while 469 rams (60.6 %) showed one or more mild (43.3 %) or severe (17.3 %) alterations. Examples of severe alterations observed during clinical evaluations are shown in Fig. 1. Only four (3.3 %) of ram lambs examined presented severe alterations. Ninety-four of the 129 rams classified as unsuitable for breeding were recommended for culling, owing to the severity of alterations. The remaining rams were recommended for treatment and not recommended for use as breeders until recuperation.

The prevalence of alterations in each body area, the prevalence of previous diseases, and the classification of rams according to age (yearlings, adults and aged rams) is summarised in Table 1. The percentage of unsuitable rams was significantly greater in aged rams than in yearlings and adults (37.4 vs. 7.9 and 13.8 %, respectively; *p* < 0.05). It was found that 16.6 % of the aged rams presented severe genital alterations (testicular alterations: 8.4 %; ulcerative posthitis: 6.9 %). The group of aged rams presented the highest prevalence of rams with testicular alterations, although significance was not attained (*p* > 0.05). The group of aged rams also displayed a greater proportion of individuals with severe alterations in head and limbs (*p* < 0.05). The only difference observed in clinical examination between yearling and adult rams was the proportion of rams free of head and genital alterations (head: 75.8 vs. 64.5 % and genitalia: 78.4 vs. 68.0 %, in yearlings and adults, respectively; *p* < 0.05).

**Table 1:** Prevalence (%) of rams with alterations and suitability of rams for breeding according to age (*n* = 774)

Body area	Lesions severity	12 – 24 months (*n* = 153)	24 – 72 months (*n* = 490)	≥72 months (*n* = 131)	TOTAL
Head, neck and trunk	None	75.8^a^	64.5^b^	41.2^c^	62.8
	Mild	22.2	32.2	34.4	30.6
	Severe	2.0^a^	3.3^a^	24.4^b^	6.6
Prognathism	None	98.0^a^	91.6^b^	84.7^b^	91.7
	Mild	2.0^a^	7.3^a^	14.5^b^	7.5
	Severe	0.0^d^	1.0	0.8	0.8
Limbs	None	93.5^a^	86.9^a^	64.9^b^	84.5
	Mild	5.9^a^	11.4^a^	25.2^b^	12.7
	Severe	0.7^a^	1.6^a^	9.9^b^	2.8
Leg conformation	None	100.0	99.0^a^	93.9^b^	98.3
	Mild	0.0^d^	1.0^a^	3.8^b^	1.3
	Severe	0.0^d^	0.0^d^	2.3	0.4
Genitals	None	78.4^a^	68.0^b^	58.0^b^	68.3
	Mild	15.7	22.2	24.4	21.3
	Severe	5.9^a^	9.8^a^	16.6^b^	10.3
Testicles	None	96.7	94.9	91.6	94.7
	Yes	3.3	5.1	8.4	5.3
Posthitis	None	91.5^a^	81.0^b^	71.0^c^	81.4
	Mild	7.8^a^	15.7^b^	22.1^b^	15.3
	Severe	0.7^a^	3.3^a^	6.9^b^	3.4
Glans	None	95.4	95.9	96.9	96.0
	Mild	3.3	2.9	3.1	3.0
	Severe	1.3	1.2	0.0^d^	1.0
Rams with any alterations	None	54.2^a^	40.0^b^	19.8^c^	39.4
	Mild	37.9	46.1	38.9	43.3
	Severe	7.8^a^	13.9^a^	41.3^b^	17.3
Previous disease	None	87.6^a^	60.2^b^	30.5^c^	60.6
	Yes	12.4^a^	39.8^b^	69.5^c^	39.4
BSE	Suitable	92.2^a^	86.1^a^	62.6^b^	83.3
	Unsuitable	7.9^a^	13.8^a^	37.4^b^	16.7

^a,b,c^Means in the same row with different superscripts differ significantly *p* < 0.05

^d^This category is not used for multiple comparison because the proportion is equal to zero

The percentages of rams with alterations in each body area by BCS (<2.5; 2.5–2.75; 3–3.5; >3.5) are summarised in Table 2. The mean age of the rams with poor BCS (emaciated; *n* = 24) was higher than that of the rams in the other three BCS groups (<2.5: 57.8 months vs. 2.5–2.75: 41.2 months; 3–3.5: 43.3 months; >3.5: 43.1 months; *p* < 0.05), without significant differences between them. Emaciated rams also presented the highest percentage of unsuitable rams, although significance was not attained (*p* > 0.05). The two groups with lower BCS showed significantly higher proportions of rams with head abnormalities (*p <* 0.05). Both groups of rams also presented significantly higher proportions of rams with prognathism than the other two groups (<2.5: 16.7 %; 2.5–2.75: 22.8 %; 3–3.5: 4.2 %; >3.5: 1.2 %; *p <* 0.05).

**Table 2:** The percentages of rams with alterations in each body area and suitability of rams for breeding by BCS (*n* = 747)

Body area	Lesions severity	<2.5 (*n* = 24)	2.5 – 2.75 (*n* = 180)	3 – 3.5 (*n* = 383)	>3.5 (*n* = 160)	Total
Head, neck and trunk	None	25.0^a^	51.7^a^	69.2^b^	66.9^b^	63.1
	Mild	58.3^a^	38.3^ab^	26.6^c^	30.0^b^	31.2
	Severe	16.7^a^	10.0^ab^	4.2^c^	3.1^b^	5.8
Prognatism	None	83.3^a^	77.2^a^	95.8^b^	98.8^b^	91.6
	Mild	16.7^a^	20.6^a^	3.9^b^	0.6^b^	7.6
	Severe	0.0^d^	2.2	0.3	0.6	0.8
Limbs	None	70.8	80.6	86.2	89.4	85.0
	Mild	25.0	18.3	10.7	10.6	13.0
	Severe	4.2	1.1	3.1	0.0^d^	2.0
Legs conformation	None	95.8	98.3	97.9	99.4	98.3
	Mild	4.2	1.7	1.3	0.6	1.3
	Severe	0.0^d^	0.0^d^	0.8	0.0^d^	0.4
Genital	None	70.8	63.3	71.5	69.8	69.2
	Mild	12.5	27.2	18.3	19.5	20.5
	Severe	16.7	9.4	10.2	10.7	10.3
Testicles	None	83.3^a^	95.0^ab^	96.1^b^	93.1^ab^	94.8
	Yes	16.7^a^	5.0^ab^	3.9^b^	6.9^ab^	5.2
Posthitis	None	83.3	80.6	82.0	83.0	81.9
	Mild	8.3	16.7	15.1	12.6	14.7
	Severe	8.3	2.8	2.9	4.4	3.4
Glans	None	95.8	93.9	97.4	95.0	96.0
	Mild	0.0^d^	4.4	1.6	5.0	2.9
	Severe	4.2	1.7	1.0	0.0^d^	1.1
Rams with any alterations	None	12.5^a^	31.7^ab^	43.9^c^	44.0^bc^	39.9
	Mild	54.2	50.0	39.7	43.4	43.4
	Severe	33.3^a^	18.3^ab^	16.4^ab^	12.6^b^	16.6
Previous disease	None	50.0	63.9	58.5	63.5	60.6
	Yes	50.0	36.1	41.5	36.5	39.4
BSE	Suitable	66.7	81.7	85.4	86.3	84.1
	Unsuitable	33.3	18.3	14.6	13.8	15.9

^a,b,c^Means in the same row with different superscripts differ significantly *p* < 0.05

^d^This category is not used for multiple comparisons because the proportion is equal to zero

The serological analyses were negative for *Brucella ovis* in all the rams.

The results of the surveys in the farms are summarised in Table 3. The most common reproduction schedule was that of three lambings in two years system, with three breeding periods per year. Only two farms used the one lambing per year system. Outside the breeding periods, rams were mainly housed in mid-covered sheepfolds, separated from ewes.

**Table 3:** General characteristics and prevalence of alterations in rams of farms visited for the clinical examinations

Variable	Min.	Perc. 25	Mean ± SEM	Perc. 75	Max.
Total number of ewes	264	530	878 ± 126	990	4770
Ram:ewe ratio	1:21	1:28	1:37 ± 1.8	1:45	1:61
Percentage of rams	1.6	2.2	2.8 ± 0.1	3.5	4.6
Suitable rams for breeding (%)	1.4	2.0	2.5 ± 0.1	2.9	3.7
Effective rams (%)	0.3	1.6	2.1 ± 0.1	2.3	3.5
Age of rams (months)	28.79	38.92	45.16 ± 1.45	50.31	67.64
Age of rams at first breeding (Months)	7	8	10 ± 0	12	18
Ewes’ turnover (%)	8.0	14.1	16.0 ± 0.6	18.0	23.0
Rams’ turnover (%)	7.0	15.0	19.5 ± 1.1	23.0	35.3
BCS (1 – 5)	2.63	2.97	3.26 ± 0.06	3.51	4.16
Prognathism	0.00	0.00	0.05 ± 0.02	0.02	0.51
Testicular alterations	0.00	0.00	0.06 ± 0.01	0.11	0.29
Genital alterations (severe)	0.00	0.03	0.12 ± 0.03	0.15	0.69
Non-genital alterations (severe)	0.00	0.00	0.08 ± 0.01	0.13	0.23
Risk of failure	0.00	0.09	0.19 ± 0.03	0.22	0.85

Risk of failure: probability of finding unsuitable rams in the flock

*Min* minimum value, *Max* maximum value, *Perc* percentile, *SEM* standard error of the mean, *BCS* body condition score

A significant positive association was found between the age of the rams and the prevalence of previous pathologies (r = 0.413; *p < 0.001*). These pathologies were mainly blowfly myiasis (96.0 %; *n* = 305); however, during the clinical examinations, only 19 rams exhibited active blowfly myiasis. A significant positive association between a history of previous blowfly myiasis and the prevalence of ulcerative posthitis (12.5 % without vs. 34.8 % with previous blowfly myiasis; *p < 0.001*) was noted.

Morphology was the most important criterion for the selection of replacement rams applied by sheep owners, followed by genealogy and growth rate until weaning. The scores of the parameters used for selection of future breeders and the main causes for culling rams are summarised in Table 4.

**Table 4:** Mean scores of the main criteria applied by sheep owners for ram lamb selection as future breeders and the main causes of elimination of rams

Criteria for selection	Mean ± SEM (1 – 5)	Causes of elimination	Mean ± SEM (1 – 5)
Morphology	4.35 ± 0.16	Age	4.71 ± 0.10
Genealogy	3.65 ± 0.28	Disease	3.47 ± 0.24
Growth until weaning	3.23 ± 0.25	Feet and leg alterations	3.44 ± 0.17
Scrapie genotype	2.90 ± 0.30	BCS	3.38 ± 0.22
Genitalia	2.64 ± 0.26	Genital alterations	2.91 ± 0.24
Consanguinity	2.13 ± 0.26	Lack of libido	2.47 ± 0.26
Libido	1.43 ± 0.12	Aggressiveness	2.32 ± 0.28

*SEM* standard error of the mean

## Discussion

In the present study, we show the results of the first phase of a programme for the evaluation of the potential fertility of rams in commercial flocks for natural breeding based on the clinical examination of the rams. To the best of our knowledge, this is the first programme performed on a large scale in Spain. In fact, previous programmes were based specifically on the evaluation of the prevalence of testicular alterations with the aim of controlling *Brucella ovis* [11, 20, 21]. Conversely, several studies have been carried out in other countries [2, 3, 5, 18, 22, 23].

The percentage of rams recorded with severe and mild alterations (60.0 %) was greater than that noted in studies performed in other breeds (34 % [2]; 48.8 % [5]). It is possible that this difference is related to the reproductive system used in farms. Those studies were performed in flocks with a low intensive reproduction management (one breeding period and one lambing per year), whilst in the sheep flocks located in the North-Eastern Spain, the main reproduction management consists of three lambings in two years (three breeding periods per year). Thus, the results of this study are derived from rams that have to perform intensive activity in a short period of time (30–35 days) every four months. In general, the most frequent alterations, independent of age and BCS, were those occurring in the head, neck and trunk or in the genitalia (mainly ulcerative posthitis and those affecting the testicles). The main genital alteration recorded in the present study was ulcerative posthitis. This type of alteration could reduce the ram’s willingness to mate because of pain when the lesion is in contact with the ewe during breeding [5, 18, 24]. A high prevalence of this alteration can thus reduce the reproductive performance of the flock. The prevalence of ulcerative posthitis might be related to the management of rams, such as their feeding or fold cleanliness. Feeding with excessively rich protein diets might lead to excessive proliferation of normally inhabitant bacteria of the prepuce such as *Corynebaterium renale* in response to the increased concentration of urea in the urine [18, 25] which favours ulcerative posthitis [26]. This type of alteration is commonly associated with preputial blowfly myiasis [26]. In fact, in the present study, 34.8 % of rams with a history of blowfly myiasis presented ulcerative posthitis during the clinical examination, whilst only 12.5 % of rams without previous blowfly myiasis did so. The percentage of rams with preputial blowfly myiasis was almost three-fold lower than results recorded by Fthenakis et al. [5], although, our data was recorded in different seasons. While our clinical examinations were performed from March to December, those in the study of Fthenakis et al. [5] were performed from May to July; therefore, taking into account that the insects that cause preputial blowfly myiasis are more active from May to October in Mediterranean countries [27], these results are not comparable. Almost half of the rams affected with preputial myiasis were located on the same farm, indicating the presence of underlying associated factors, such as either environmental factors or those related to the management of rams. Another cause of ulcerative posthitis that might not be disregarded is contagious ecthyma [10, 25], which was not evaluated in the present study.

In the present study, the occurrence of alterations was closely related to the age of males. In this way, mature rams (adult and older rams) presented a higher proportion of unsuitable rams. In fact, only 19.8 % of the aged rams evaluated were free of alterations. This has also been reported in other studies [5, 18], in which both groups, adult and aged rams, presented a higher proportion of affected individuals. The percentage of rams with testicular alterations (i.e., orchitis, epididymitis, atrophy or hypoplasia) was lower than in other studies [2, 5, 18]. These good results could be a consequence of previous health actuations performed on farms to reduce the prevalence of *B. ovis* [11]. As noted by Van Metre et al. [18], the groups of mature rams (adult and aged rams) also presented higher percentages of testicular alterations. These results are especially noteworthy, as testicular alterations could have adverse effects on semen quality and on the libido of rams [5]. In spite of the low general prevalence of limb alterations, higher percentages were observed in aged rams than in the other three age groups. This would indicate that an increased amount of foot care is required as rams get older [2].

The physical condition of the males was another determinant of the presence of alterations. The most affected were emaciated males (BCS < 2.5), which presented the highest proportion of rams with severe alterations in the head, limbs and genital areas; moreover, those rams were much older than in the other three BCS groups. These results indicate that the loss of BCS could be indicative of disease or of significant alterations [3, 18]. Nevertheless, these results should be considered carefully owing to the low number of individuals in the group of emaciated rams.

Both, the group of emaciated rams and the group of rams with BCS 2.5–2.75 involved a greater proportion with prognathism (the rams with marked prognathism were recommended for culling). This type of alteration affects the correct relationship of the dental pad and the lower teeth upon closure. These results suggest that prognathism would lead to poor BCS, owing to greater difficulty in food intake.

It was striking that the group of rams found with BCS 2.5–2.75 (24.1 %; 180 out of 747 rams) had such a poor BCS in spite of their young mean age, similar to the groups of higher BCS (3–3.5 and >3.5). The main causes for that finding might be a lower hierarchy, wrong feeding management or underlying disease.

All of the rams examined were seronegative for *B. ovis*. These results were similar to previous findings recorded in studies carried out from 1998 to 2006 [11]. Conversely, higher percentages of seropositive rams have been reported in other countries [18]. The low prevalence observed in our study might be a consequence of previous sanitary programmes made in farms to eliminate males with testicular alterations [20], mainly the official vaccination programmes against *B. melitensis (Rev-1),* which also protect against *B. ovis* [28, 29].

The mean ram:ewe ratio in flocks was 1:37 (2.8 %). Similar results were recorded in Greece by Fthenakis et al. [5]; however, comparisons must be made carefully owing to the different production management used in both regions (1 breeding period per year vs. 3 breeding periods per year). For example, breeding during the anoestrous period using hormonal treatments increases the requirement of ram numbers during breeding [30, 31]. If only suitable breeders were taken into account, the mean ram:ewe ratio would fall to 1:42 (percentage of effective rams: 2.5 %). The proportion of effective rams in several flocks was much lower, which could negatively affect flock fertility [32].

The mean age of rams was similar to that reported by Fthenakis et al. [5], with a high variability between flocks. As shown in Table 3, the main cause of this variability might be the different replacement rate of rams in farms. For example, some farms had introduced only a few new breeders in the previous two years, while others had introduced a large number of new breeders (ram lambs). The mean turnover percentage of rams was 19.5 ± 1.5 %, which was always greater than the mean turnover of ewes (16.0 ± 0.6 %). These results are close to recommendations in the literature (20 %), which could reach up to 30 % depending on the flock characteristics [14]. Using incorrect replacement rates for rams could lead to an aged ram population or to an excessive proportion of ram lambs and yearlings, which have lower potential fertility than adults [2, 33].

The mean age of rams at first breeding was 10 months, which was lower than the value observed by Uthlaut et al. [19]. In some farms, the age at first breeding was markedly higher. These farms had higher proportions of rams of foreign origin, indicating that sheep owners prefer buying yearlings to ram lambs.

The risk of failure was highly variable (Table 3), ranging from 0.0 to 85.0 %. These results corroborate the findings reported by Fthenakis et al. [5]. This variability is probably due to factors associated with the environment, sanitary control and the management applied in each flock. For example, in one flock, 50 % of the males were affected by prognathism. This could be indicative of poor criteria during the selection of ram lambs as future breeders. Nevertheless, as the proportion of rams with prognathism was greater in older rams than in younger ones, it could be interpreted that greater care is currently being applied in the selection of replacement rams.

The survey of sheep owners showed that the main criteria applied to ram lambs selection as future breeders were morphology, genealogy and growth rate until weaning. Morphology was the most important criterion, and libido was almost irrelevant. Uthlaut et al. [19] reported similar results, although the scrapie resistance genotype was considered less important in our study. The lack of importance of the libido in the selection of ram lambs is reasonable taking into account that lamb selection is performed before puberty, at 2.5–3 months of age and that detecting rams with consistently low libido is not an easy task. As previously reported by Uthlaut et al. [19], the main reason for culling rams was their advanced age. However, the number of rams culled owing to low libido and genital issues was considered of little importance. Thus, taking into account that 10 % of rams presented severe alterations in the genitalia, a higher frequency of genital examinations is recommended in order to detect and cull these rams.

## Conclusion

The results reported here were recorded in the first phase of a new programme based on the clinical examinations of rams, which was implemented for the improvement of flock fertility. Aged rams (older than 72 months) presented higher percentages of unsuitability, which might lead to poor fertility rates. Therefore, rams exceeding that age should be considered for culling. Several farms apply inadequate turnover percentages which lead to increased mean ram age. Although the mean BCS was close to adequate values, some farms presented excessively low or high values indicating that feeding management needs to be revised. We conclude that there is a lower proportion of effective rams than sheep owners believe. More frequent examinations are therefore required to detect and treat unsuitable breeders. The detection of rams needing treatment and potentially infertile rams should be prioritised. This information should be utilised by sheep owners to schedule the turnover of rams. Finally, the effectiveness of the present programme to increase the productivity of flocks has to be assessed in the coming years, and the introduction of other tests for semen quality and libido assessment (serving capacity) should be considered provided that their procedure can be simplified.

## Abbreviations

BCS: body condition score
BSE: breeding soundness evaluations
CITA: Centro de Investigación y Tecnología Agroalimentaria de Aragón
Max: maximum value
Min: minimum value
NE: North-Eastern
Perc: percentile
RA: Rasa Aragonesa
SCT: serving capacity tests
SEM: standard error of the mean
WOAH: World Organisation for Animal Health
